# Identifying novel host-based diagnostic biomarker panels for COVID-19: a whole-blood/nasopharyngeal transcriptome meta-analysis

**DOI:** 10.1186/s10020-022-00513-5

**Published:** 2022-08-03

**Authors:** Samaneh Maleknia, Mohammad Javad Tavassolifar, Faezeh Mottaghitalab, Mohammad Reza Zali, Anna Meyfour

**Affiliations:** 1 Basic and Molecular Epidemiology of Gastrointestinal Disorders Research Center, Research Institute for Gastroenterology and Liver Diseases, Shahid Beheshti University of Medical Sciences, Tehran, Iran; 2 Gastroenterology and Liver Diseases Research Center, Research Institute for Gastroenterology and Liver Diseases, Shahid Beheshti University of Medical Sciences, Tehran, Iran

**Keywords:** COVID-19, Biomarker, Data integration, Systems biology, Whole blood, Nasopharyngeal swab, SARS-COV-2, Random forest, Pathogenesis

## Abstract

**Background:**

Regardless of improvements in controlling the COVID-19 pandemic, the lack of comprehensive insight into SARS-COV-2 pathogenesis is still a sophisticated challenge. In order to deal with this challenge, we utilized advanced bioinformatics and machine learning algorithms to reveal more characteristics of SARS-COV-2 pathogenesis and introduce novel host response-based diagnostic biomarker panels.

**Methods:**

In the present study, eight published RNA-Seq datasets related to whole-blood (WB) and nasopharyngeal (NP) swab samples of patients with COVID-19, other viral and non-viral acute respiratory illnesses (ARIs), and healthy controls (HCs) were integrated. To define COVID-19 meta-signatures, Gene Ontology and pathway enrichment analyses were applied to compare COVID-19 with other similar diseases. Additionally, CIBERSORTx was executed in WB samples to detect the immune cell landscape. Furthermore, the optimum WB- and NP-based diagnostic biomarkers were identified via all the combinations of 3 to 9 selected features and the 2-phases machine learning (ML) method which implemented k-fold cross validation and independent test set validation.

**Results:**

The host gene meta-signatures obtained for SARS-COV-2 infection were different in the WB and NP samples. The gene ontology and enrichment results of the WB dataset represented the enhancement in inflammatory host response, cell cycle, and interferon signature in COVID-19 patients. Furthermore, NP samples of COVID-19 in comparison with HC and non-viral ARIs showed the significant upregulation of genes associated with cytokine production and defense response to the virus. In contrast, these pathways in COVID-19 compared to other viral ARIs were strikingly attenuated. Notably, immune cell proportions of WB samples altered in COVID-19 versus HC. Moreover, the optimum WB- and NP-based diagnostic panels after two phases of ML-based validation included 6 and 8 markers with an accuracy of 97% and 88%, respectively.

**Conclusions:**

Based on the distinct gene expression profiles of WB and NP, our results indicated that SARS-COV-2 function is body-site-specific, although according to the common signature in WB and NP COVID-19 samples versus controls, this virus also induces a global and systematic host response to some extent. We also introduced and validated WB- and NP-based diagnostic biomarkers using ML methods which can be applied as a complementary tool to diagnose the COVID-19 infection from non-COVID cases.

**Supplementary Information:**

The online version contains supplementary material available at 10.1186/s10020-022-00513-5.

## Introduction

Severe acute respiratory syndrome coronavirus 2 (SARS-COV-2), known as 2019-nCoV, has spread worldwide, causing about 490 million cases and more than 6.15 million fatalities (5 April 2022). Coronavirus disease 2019 (COVID-19) has a wide range of clinical manifestations, from asymptomatic patients to severe inflammatory reactions, resulting in organ failure and death (Chen et al. 2020). It is not yet completely clarified whether severe outcomes of disease are related to the viral infection, the host's immunological response, host underlying diseases, or a combination of these variables (Williamson et al. 2020). Evidence indicates that the host responses to SARS-COV-2 are highly different than antiviral responses to other respiratory viruses like influenza and seasonal corona (Smith et al. 2021). According to the analysis of SARS-COV-2-specific immunological responses, SARS-COV-2 inhibits innate immune system activation like dendritic cells while increasing proinflammatory macrophage activation and IL-6 and tumor necrosis factor (TNF) production (Zhou et al. 2020; Schultze and Aschenbrenner 2021). Besides, studies have revealed that one of the important damaging factors, locally in the lungs and systemically in the circulation, is an increase in neutrophils (Vanderbeke et al. 2021). Despite the observed peripheral lymphopenia, SARS-COV-2-specific B cell responses and the number of plasma cells increase in COVID-19 patients (Smith et al. 2021).

Recent studies have demonstrated that the host transcriptome undergoes substantial changes upon SARS-COV-2 infection in a variety of tissues like respiratory epithelial cells, nasopharynx, colonocytes, and whole blood or plasma samples (Ong et al. 2020; Lioa et al. 2020). Therefore, RNA sequencing can be employed as a robust tool to identify host transcriptional signatures affected by SARS-COV-2, leading to the development of some novel diagnostic biomarkers and therapeutic strategies (Ng et al. 2021; Thair et al. 2020a; Mick et al. 2020). Furthermore, understanding the similarities and differences of the host response to SARS-COV-2 infection compared to other (viral or non-viral) respiratory infections is necessary to find common/virus-specific transcriptional signatures (Thair et al. 2020a).

The conventional diagnostic test for COVID-19, viral nucleic acid amplification tests (NAAT) using reverse transcription-polymerase chain reaction (RT-PCR), provides a considerable rate of false-negative results because the virus load in individuals can be low and significantly change during the course of the disease (Pan et al. 2020; Wölfel et al. 2020). Therefore, identification of the host-specific biomarkers as a complementary tool is critical to accurately diagnose the COVID-19 infection from non-COVID cases (Mick et al. 2020). Several studies focused on finding effective repurposable drugs for the COVID-19 treatment and introduced some potential therapeutic targets by analyzing a single gene expression dataset of COVID-19 patients. In a study by Ahmed et al., a microarray dataset consisting of 10 and 4 PBMC samples of patients with SARS-CoV-1 infection and healthy controls, respectively was analyzed to find hub genes and remarkable signaling pathways in SARS-CoV-1 infection. The repurposable drugs for SARS-CoV-1 infections were then identified and validated for the treatment of SARS-CoV-2 infections (Ahmed et al. 2022). Furthermore, Mosharaf et al. analyzed an RNA-seq dataset consisting of 35 lung tissue samples infected with SARS-COV-2 (case) and 5 control samples to find differentially expressed genes (DEGs). By constructing a protein–protein interaction network, key genes and signaling pathways in SARS-COV-2 infection were determined to be used as targets for drug repurposing in COVID-19 (Mosharaf et al. 2022). However, recent developments in "omics" technologies, along with advances in computer sciences, have provided an opportunity to integrate and analyze multi-cohort datasets using systems biology approaches and machine learning (ML) methods, leading to decreased heterogeneity of publicly available single population-based transcriptome datasets (Tavassolifar et al. 2020). The multi-cohort analysis of published transcriptional data derived from whole blood (WB), peripheral blood mononuclear cells (PBMCs), epithelial cells, or cell lines that represented infections from 7 viruses (adenovirus, influenza, SARS, RSV, HRV, enterovirus, HHV6) and 4 bacteria (S. pneumonia, S. aureus, E. coli, Salmonella), proposed a common host signature across different respiratory viral infections. These findings could discriminate individuals with viral infections from those with bacterial infections and healthy controls (Andres-Terre et al. 2015).

**Fig. 1:**
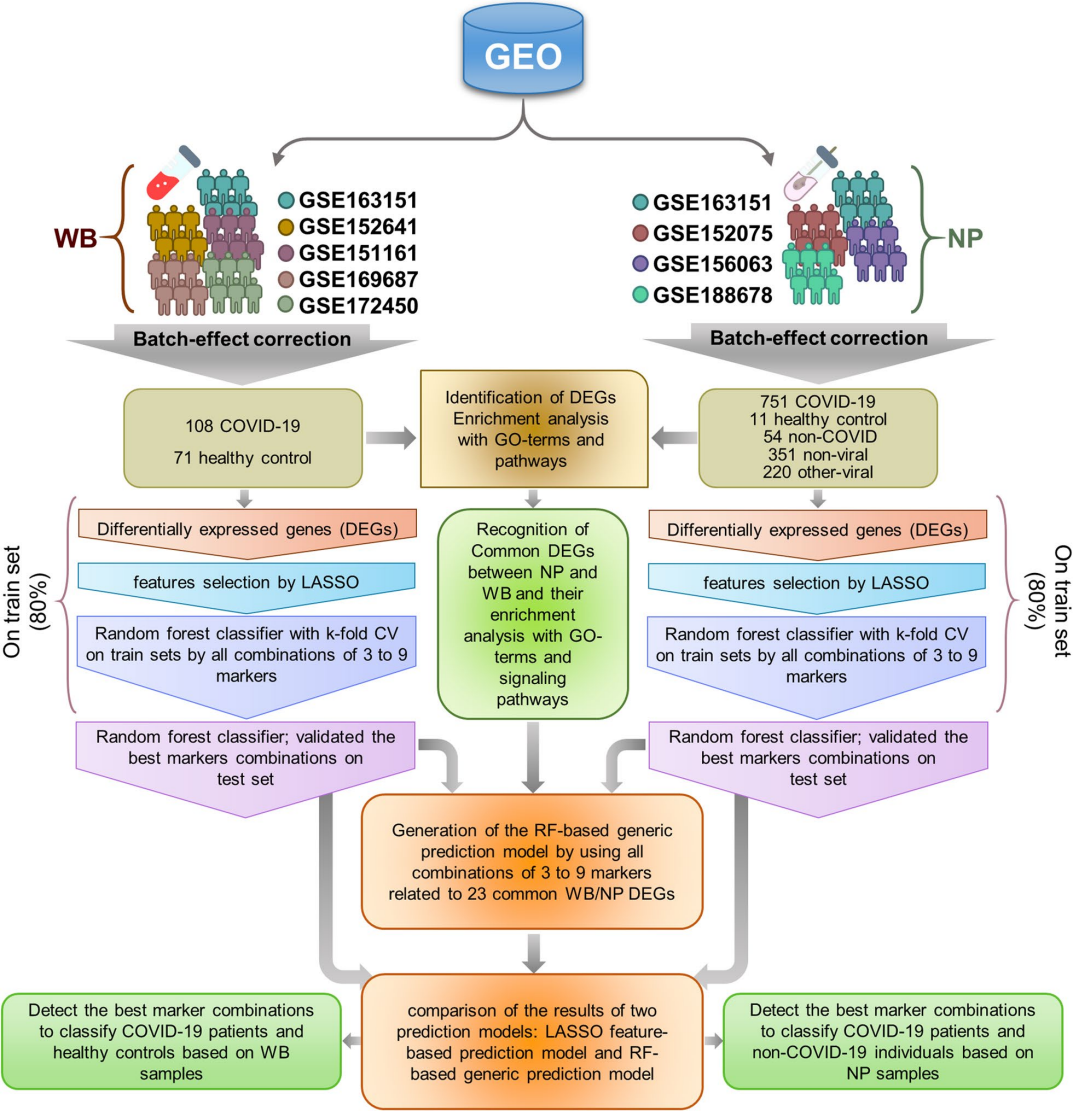
The workflow of the study: The RNA-Seq datasets related to whole blood (WB) and nasopharyngeal (NP) samples from patients with COVID-19 infection and other similar disease conditions including viral and non-viral acute respiratory illnesses (ARI) as well as healthy controls were acquired from GEO database. Data were integrated and the batch effects were eliminated. Subsequently, the datasets were subjected to pathway enrichment and GO analyses. Furthermore, the candidate diagnostic biomarker panels were identified using machine learning methods on train datasets and validated on independent cohorts to introduce the best biomarker combinations. Besides, the RF-based generic prediction models were generated by using all combinations of 3 to 9 markers related to 23 common WB/NP DEGs was done. Finally, the results of two prediction models, including the LASSO feature-based prediction model and RF-based generic prediction model were compared. *WB* whole blood, *NP* nasopharyngeal, *ARI* acute respiratory illnesses, *RF* random forest

Cross-platform normalization (CPN) as a data integration technique increases sample sizes, improves overall heterogeneity estimation, identifies more specific host response signatures, and reduces the effect of individual study-specific biases. It has been shown that novel diagnostic panels with more power, robustness, and generality can be introduced by using CPN (Hamid et al. 2009; Larsen et al. 2014; Irigoyen et al. 2018; Taminau et al. 2014; Maleknia et al. 2020). In this study, we developed an integrated, multi-cohort analysis framework that takes advantage of the heterogeneity seen in Gene Expression Omnibus (GEO) public data repositories to identify and validate robust, reproducible, and more specific host response signatures by CPN. This study involved the five gene expression datasets including 179 human WB samples from SARS-COV-2 infected patients and healthy controls (HC) and also four gene expression datasets including 1387 human nasopharyngeal (NP) samples from patients with SARS-COV-2 infection, other viral acute respiratory illnesses (ARIs), and non-viral ARIs, as well as HC individuals. Our methods were employed for two different hypotheses. In the first step, the gene expression profiles of 9 WB and NP datasets were analyzed to find out SARS-COV-2 pathogenesis and perturbed host immune response pathways which we termed ‘COVID-19 meta-signature’ (CMS). We investigated the similarities and differences of the host response to SARS-COV-2 infection compared to other (viral or non-viral) respiratory infections in NP samples. In the continuum, to introduce diagnostic biomarker panels as complementary tools along with the conventional diagnostic test, we leveraged feature selection and ML methods on the integrated datasets. In the first phase of the ML methods, the optimum combinations of features were designated by using k-fold cross validation on a train set (80% of the population), and then in the second phase, the best combinations based on accuracy parameter were selected to be validated on independent test sets (20% of the population). Finally, we could identify high-performance 3 to 9-biomarker panels related to WB and NP samples that could accurately distinguish COVID-19 patients from HCs and non-COVID individuals (Fig. 1).

## Methods

### Data collection and pre-processing

The entire analysis was accomplished with publicly available data. In order to reach proper gene expression profiles, the keyword “COVID-19” was searched in the GEO database restricted to “Homo sapiens” taxonomy and “Expression profiling by high throughput sequencing”. Afterwards, data obtained from WB and NP samples were selected, while the data related to organoids, single-cell, cell lines, recurrent patients, and drug treatments were excluded from the results. Finally, by setting the sample count on more than 20 samples on 11/20/2021, three datasets with accession numbers GSE163151, GSE151161, and GSE152641 related to WB samples and four datasets with accession numbers GSE163151, GSE152075, GSE156063, and GSE188678 related to NP samples were used for this study. To raise the number of HCs related to WB, 27 HC samples were imported to the study from two non-COVID studies with accession numbers GSE169687 and GSE172450 (Table 1).

**Table 1:** The number of WB and NP samples applied in this study

Tissue	GSE^a^	COVID-19	HC	Not-COVID	Other respiratory diseases	Refs
WB	163,151	7	20			Ng et al. (2021)
WB	151,161	39				–
WB	152,641	62	24			Thair et al. (2020a)
WB	169,687		14			–
WB	172,450		13			–
NP	152,075	430		54		Lieberman et al. (2020)
NP	156,063	93			Other viruses = 41, non-viral = 100	Mick et al. (2020)
NP	163,151	138	11		Influenza = 76^b^, other Corona viruses = 12^b^, other viruses = 32, non-viral = 82	Ng et al. (2021)
NP	188,678	90			other viruses = 59, non-viral = 169	–

^a^Genomic Spatial Event (GSE) database (Danford et al. 2008)

^b^The samples related to “Influenza” and “other corona” were labeled as other viruses in the analysis

Rather than doing conventional biological experiments, we chose cohorts from various centers to incorporate technical variation and population-related biological heterogeneity in the study. The samples in these datasets characterized different biological conditions including viral infections (SARS-COV-2, influenza, other Coronaviruses, and other viruses causing ARIs), non-viral ARIs (types of bacterial infections), and HC. To add on the technological heterogeneity property in our study, datasets profiled by RNA-seq technology from several manufacturers were used. The Ensemble IDs that represented transcripts in each dataset were mapped to gene symbols (HGNC) to facilitate integrated analysis. If multiple Ensemble IDs were matched to one gene, the expression values for those IDs were averaged to one value using the *aggregate* function from the *stats* package version 4.0.3 (Fig. 1).

We selected 9 datasets (GSE163151(WB and NP), GSE151161, GSE152641, GSE169687, GSE172450, GSE152075, GSE156063, and GSE188678). The NP samples in GSE163151 related to “Influenza” and “other corona” viruses were labeled as other viruses in the CMS analysis. Cohorts related to NP samples contained samples from SARS-COV-2 infection and other conditions such as HC, other viral ARIs, and non-viral ARIs. Cohorts related to WB samples contained samples from SARS-COV-2 infection and HC individuals.

### Data integration via cross-platform normalization

we performed the CPN method on the finalized cohorts and samples (Walsh et al. 2015). In order to gain a uniform dataset associated with each tissue while employing more heterogeneity in the population, the datasets related to each group were integrated by merge function from the base package, R 4.0.3. The genes with 0 read count in at least 60% of all classes (COVID-19, HC, non-viral, and other viral) were excluded from the datasets. Subsequently, the batch correction was performed using *ComBat-seq* from the *sva* package version 3.38 (Zhang et al. 2020) to minimize experimental variance. The parameter group was set to the vector of the disease condition (Pei et al. 2021).

### Differential gene expression analysis between COVID-19 and non-COVID

Two uniform gene expression datasets included 179 human WB samples from COVID-19 patients and HC individuals and 1387 human NP samples from patients with SARS-COV-2 infection, other viral ARIs, non-viral ARIs, and HC individuals were imported for differential gene expression analysis. Read counts were normalized by TMM scaling from the DEseq2 and edgeR packages version 1.30.1 and 3.32.1. Afterward, DEGs were identified by using the *lmFit*, and empirical Bayes (eBayes) functions within the *limma* package version 3.46.0 (Ritchie et al. 2015). The *P* values were corrected for multiple comparisons by the Benjamini–Hochberg false discovery rate (FDR) method (Benjamini and Hochberg 1995). The significant DEGs were defined by adjusted *P*-value < 0.05, and the absolute value of the log2-transformed fold-change in the expression level was set to more than 1. The volcano plots related to each set of DEGs were plotted by *ggplot2* package version 3.3.3.

### Identifying CMS by gene set enrichment and gene ontology analyses in WB and NP samples

To identify enriched biological processes and pathways associated with each set of DEGs in WB and NP samples, we utilized *EnrichR* (Kuleshov et al. 2016) and *ToppGene* (Chen et al. 2009) to explore the most significant Gene Ontology Biological processes (BPs), Molecular functions (MFs), Cellular components (CCs), and signaling pathways. Due to the popularity of the *EnrichR* tool in scientific resources (Stephenson et al. 2021; Sajuthi et al. 2020; Unterman et al. 2022; Müller et al. 2021), the results and discussion of this study are mostly based on *EnrichR* findings. We illustrated the results by dot-plot and bar plot functions in the *ggplot2* package.

### Estimation of the immune cell type proportion in WB samples by CIBERSORT

In this step, WB row counts achieved from batch correction were normalized using the counts per million (CPM). A computational method called CIBERSORTx (https://cibersort.stanford.edu/) method was applied to quantify immune cell-type proportions from Gene Expression Profiles (GEPs) (Newman et al. 2015). As reference gene expression signatures, the standard LM22 signature matrix was leveraged to estimate the relative proportions of each cell type (Chen et al. 2018). The signature matrix consists of 547 genes that precisely recognize 22 functionally defined human immune subsets, including seven T cell types, naïve and memory B cells, plasma cells, NK cells, and myeloid subsets. By applying the independent samples T-test, the differences of each cell type between two considering groups of COVID-19 patients and HC individuals were tested.

### Comparison of SARS-COV-2-infected WB and NP transcriptome profiles

In order to detect similarities and differences in gene expression patterns between two types of SARS-COV-2-infected tissues, we compared two sets of DEGs between COVID-19 patients versus HC in WB and NP samples. We first explored the DEGs sets via the venn diagram by *venn* package version 1.10 to recognize the overlapping genes between two DEGs sets. Then, enriched biological processes and pathways associated with each set of the common DEGs were obtained by *Enrichr* and *ToppGene* databases.

### In-silico discovery of diagnostic biomarkers in WB and NP samples

To discriminate COVID-19 patients from HC individuals in WB samples, an ML method was used to recognize the diagnostic biomarker panel. The same approach was applied to distinguish patients with COVID-19 and non-COVID in NP samples. Non-COVID samples in NP mean the total samples of non-viral, other viral, and the not-COVID samples in GSE152075. Samples were stratified randomly but proportionally assigned into a training set (80%) or an independent test set (20%). The methods used are as follows:

### Feature selection in the train sets of WB and NP samples

Diagnostic features were selected in two ways. Initially, the DEGs were found by pairwise comparisons among considering groups in the train sets of WB and NP samples. These genes were exploited as input features to fit a Least Absolute Shrinkage and Selection Operator (LASSO) regression model for feature selection (Tibshirani 1997; L’Heureux 2017). The *glmnet* package version 4–1.1 was used plus the cross-validation method to estimate *lambda's* regularization parameter by *cv.glmnet* function. The eligibility criteria to enter the features into the classification step were as follows: absolute LASSO coefficient more than 0.1 OR the non-zero LASSO coefficient and the absolute value of logFC more than 1.3. Subsequently, *EnrichR* and *ToppGene* databases were used to explore enriched biological processes and pathways associated with these features. In parallel, we also considered the common DEGs between NP and WB to check whether they could also be applied as diagnostic features.

### Discrimination of COVID-19 patients from non-COVID individuals by optimal biomarker panels

To detect the optimal, powerful and robust diagnostic biomarker panel obtained by the LASSO method, a two-phase ML platform was done. The Random Forest (RF) classifier (Breiman 2001) by *randomForest* R package version 4.6–14 was employed in both phases. In the first phase, the classifier was performed on the training set (80%) of the WB and NP datasets using fivefold and tenfold cross validations, respectively. The input features of the first phase were all combinations of 3 to 9 selected features based on LASSO. The statistical parameters; sensitivity, specificity, and accuracy, were estimated by *confusion Matrix* function from *caret* package version 6.0–86. The best combinations considering the highest sensitivity and specificity were picked out for the next phase. In the second phase, the best combinations of features in the training set (80%), were validated once more, based on the independent test set (20%). The sensitivity, specificity, and accuracy obtained from the two phases were displayed by line plots. Likewise, the receiver operating characteristic (ROC) curves were plotted, and the Area Under Curve (AUC) was computed through *pROC* package version 1.17.0.1.

In parallel, to find the best diagnostic panels among common DEGs between WB and NP samples of COVID-19 patients compared to HC, we used RF classifier on all combinations of 3 to 9 common features in the training set (80%), and then they were validated based on the independent test set (20%). Finally, the results of the LASSO feature-based prediction model and common WB/NP feature-based model were compared.

## Results

### Data integration and batch effect correction in WB and NP datasets

The statistics of the samples used for this study is demonstrated in Table 1. The number of WB samples for COVID-19 patients and HC individuals was 108 and 71 samples, respectively. Meanwhile, the number of NP samples for COVID-19 patients, HC, and AIRs were751, 11, and 571, respectively.

Both genders were included in these datasets, and all individuals were adults. The gene expression datasets related to each tissue were integrated, and the effect of batches was corrected to obtain a uniform dataset in WB and NP samples, individually. To check the uniformity of the datasets, principal components analysis (PCA) was performed before and after removing the batch effect (Additional file 1: Figs. S1 and S2).

### WB transcriptome analysis of patients with COVID-19

**Fig. 2:**
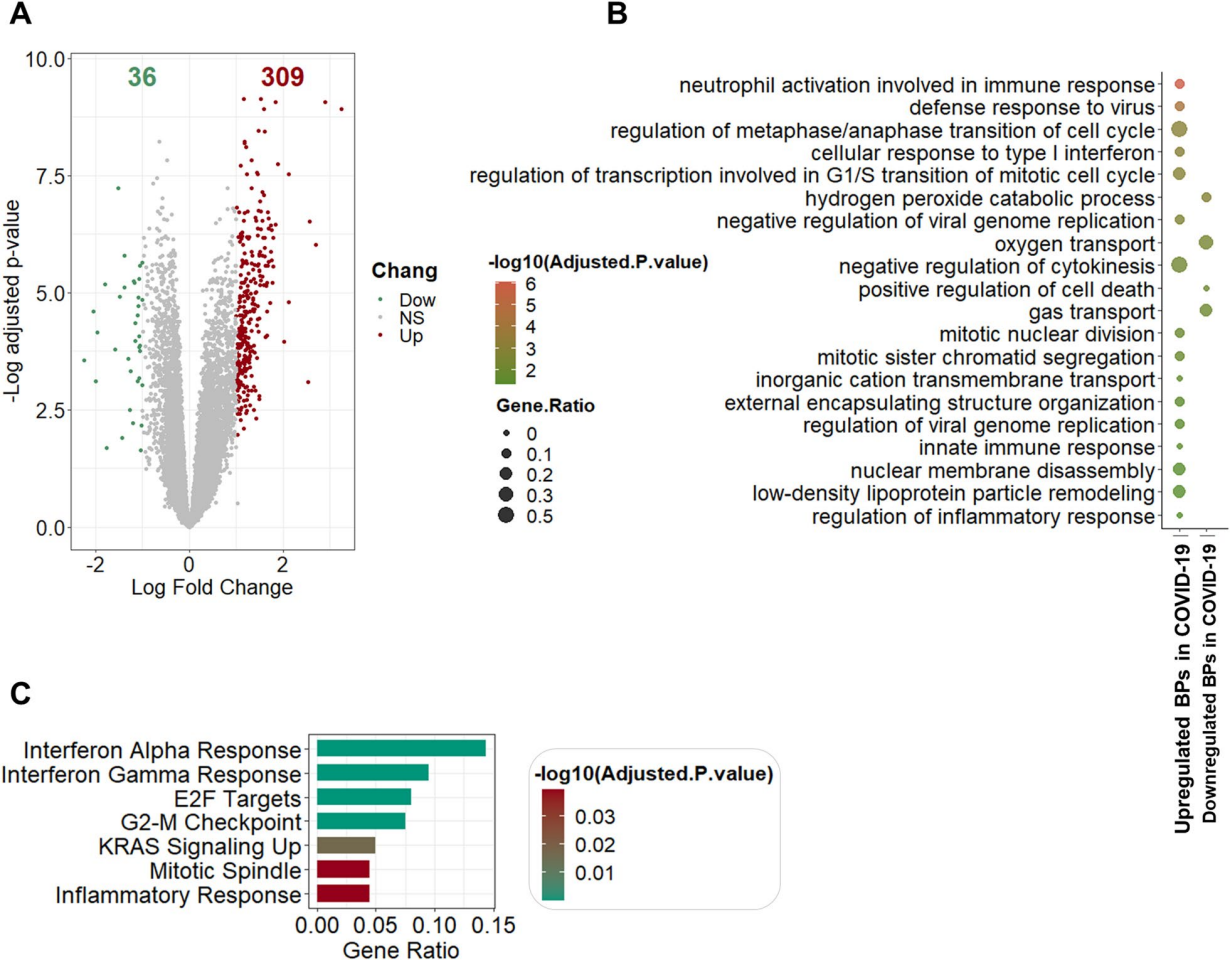
Transcriptome analysis of whole blood samples of COVID-19 patients versus healthy controls: The volcano plot to demonstrate differential expressed genes which had adjusted *P*-value < 0.05, |Log_2_FC|> 1. Red and green show up and downregulated genes, respectively (**A**). Dot plot to show BPs (GO) according to significantly upregulated and downregulated genes. The size of the dots is proportional to the gene ratio in considering process and the color corresponds to the –log10 of the adjusted *P*-value. Selected top and not-redundant terms are visualized (**B**). Bar plot to depict hallmark gene set enrichment analysis. The size of the bars is proportional to the gene ratio in considering pathway and the color corresponds to the –log10 of the adjusted P-value (**C**). *BP* biological process, *GO* gene ontology

The differential gene expression analysis between COVID-19 and HC groups in WB final dataset resulted in the identification of 345 DEGs, of which 309 genes were upregulated, and 36 genes were downregulated in COVID-19 (Fig. 2A). The 20 non-redundant significant BPs and all seven significant hallmark pathways with adjusted *P*-value < 0.05 based on *EnrichR* and *ToppGene* findings were depicted in Fig. 2B, C. The significant BPs were enhancement in “neutrophil activation”, “cell cycle”, “inflammatory host response”, “interferon signature”, and reduction in “gas and oxygen transport”. Interestingly, GO functional analyses of DEGs using the ToppGene database also confirmed that BPs listed above are highly linked with the SARS-COV-2 infection (Additional file 2: Table S1). Furthermore, downregulated genes were significantly involved in “ferrous iron binding”, “heme binding”, and “hemoglobin alpha binding” and belonged to the endocytic vesicle lumen. The pathway analysis of WB samples from patients with COVID-19 compared with HC showed notable enrichment of genes in multiple pathways associated with “inflammatory response”, “interferon-alpha”, “G2M checkpoint”, “mitotic spindle”, and “interferon-gamma response” as well as “KRAS Signaling Up”. The complete list of DEGs, significant BPs, MF, and CCs, and enriched signaling pathways based on *EnrichR* and *ToppGene* databases as well as their commonalities are available in Additional file 2: Table S1.

### COVID-19 immune cell landscape

**Fig. 3:**
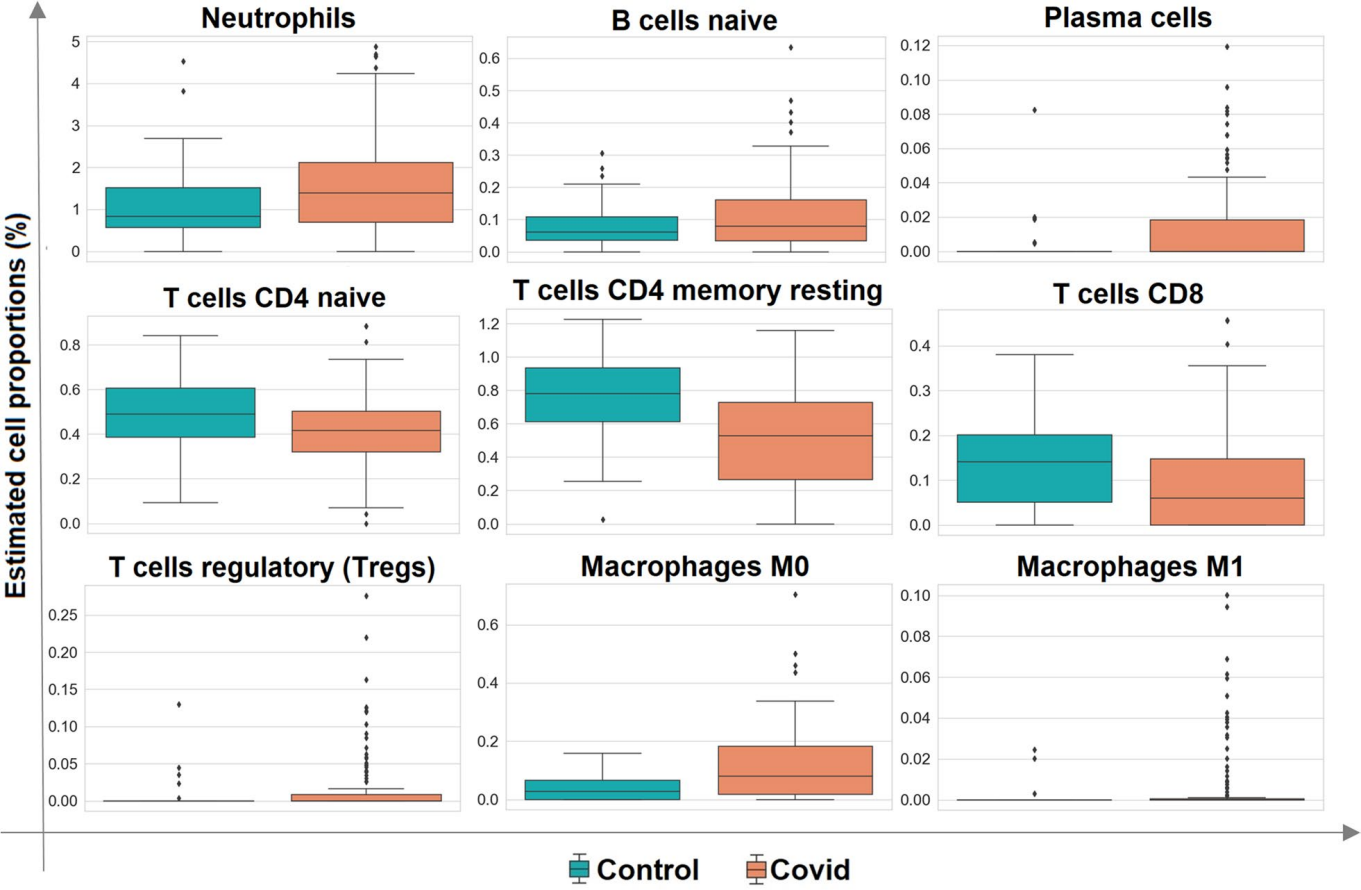
Cell-type proportions in whole blood of COVID-19 in comparison to healthy control: the box plots for the estimated immune cell type proportions of the COVID-19 patients and the HC individuals which were obtained by Cibersortx. *HC* healthy control

Specific alterations in gene expression between SARS-COV-2 and HC individuals might be derived from changes in tissue cellular composition such as immune cell types. To examine this, the CPM of WB samples was imported to the CIBERSORTx tool and cell-type proportions were estimated. The results of the independent T-test between different immune cell types of COVID-19 and HC groups were presented in Additional file 3: Table S2. Remarkably, SARS-COV-2 infection increased the proportion of T regulatory cells (Tregs) while decreasing the proportions of CD8, CD4 naïve, and CD4 memory resting cells. Correspondingly, the proportions of neutrophils, B cells naïve, plasma cells, and macrophages (M0 and M1) were augmented in COVID-19 vs HC (Fig. 3).

### NP transcriptome analysis of patients with COVID-19

To delineate the molecular pathogenesis of SARS-COV-2 based on host NP gene expression, differential gene expression, GO and hallmark gene set analyses were performed in three paired conditions including; COVID-19 vs HC, COVID-19 vs non-viral ARIs, and COVID-19 vs other viral ARIs to highlight the contrasts.

**Fig. 4:**
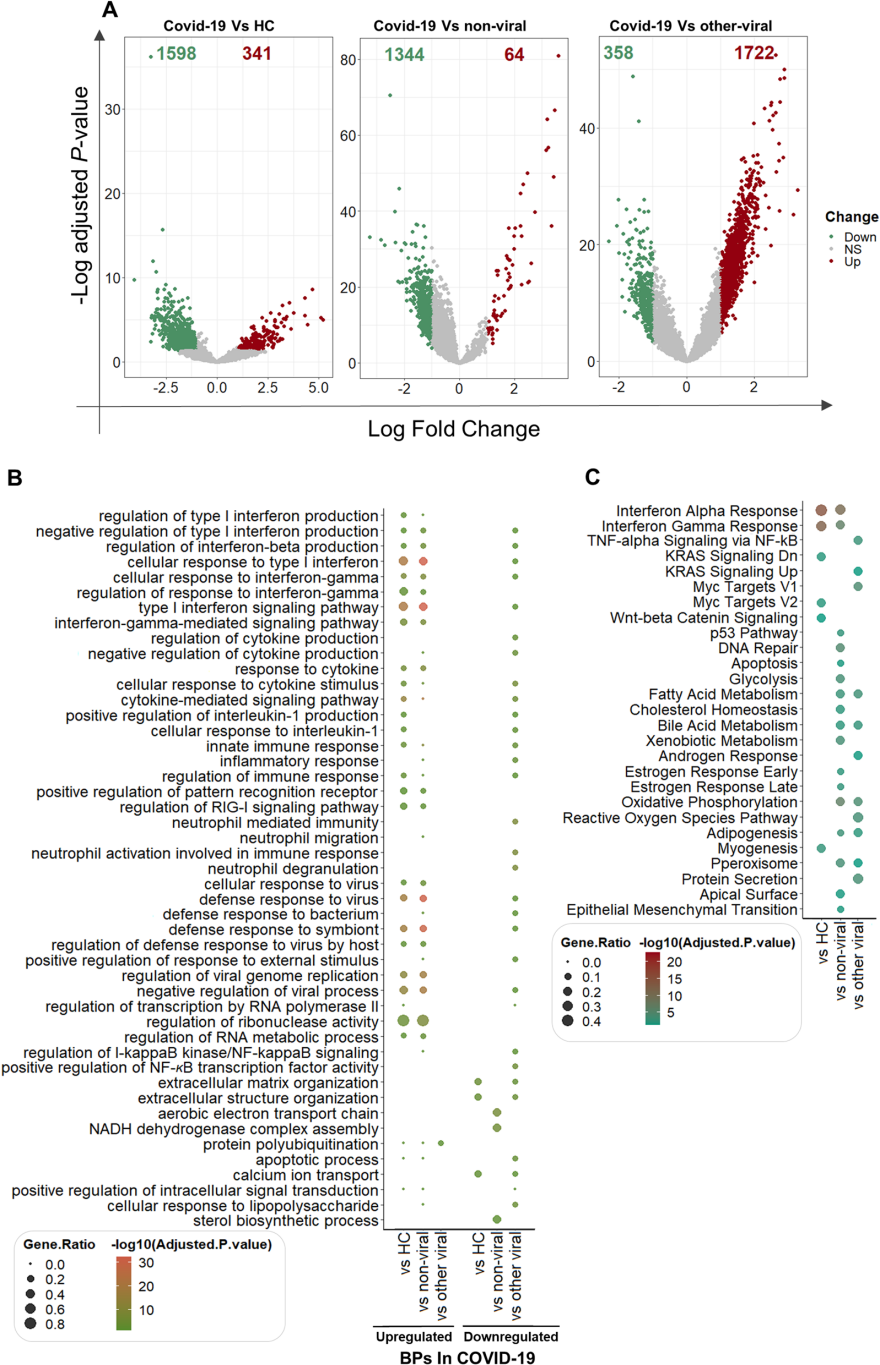
Transcriptome analysis of nasopharyngeal samples of patients with COVID-19 versus non-viral and other viral acute respiratory illnesses (ARIs) as well as healthy controls: The volcano plot to demonstrate differential expressed genes which had adjusted *P*-value < 0.05, |Log_2_FC|> 1. Red and green show up and downregulated genes, respectively (**A**). Dot plots to show BPs according to significantly up/downregulated genes (**B**) and hallmark gene set enrichment analysis (**C**). The size of the dots is proportional to the gene ratio in considering process and pathway; and the color corresponds to the –log10 of the adjusted *P*-value. Selected top and not-redundant terms are visualized. *BP* biological process

The number of up and downregulated genes were depicted in volcano plots (Fig. 4A). 341 upregulated and 1598 downregulated genes were found when the NP transcriptome profile of COVID-19 patients was compared to that of healthy individuals. The expression of 64 and 1344 genes was also significantly increased and decreased in COVID-19 compared to non-viral ARIs, respectively. Additionally, 1722 and 358 genes were up and downregulated in COVID-19 vs other viral ARIs, respectively. A striking contrast highlighted by differential gene expression analysis between the first two groups and the third group emerged in the enrichment of interferon signature. According to enrichment analysis using two databases, SARS-COV-2 infection in comparison with HC and non-viral ARIs leads to the significant upregulation of genes associated with regulation of cytokine production, cellular response to cytokines, defense response to the virus, innate immune response, and inflammatory response. However, these BPs in SARS-COV-2 infection were noticeably attenuated compared to other viral ARIs. Likewise, the genes relevant to neutrophil functions had distinct expression patterns among study groups. The expression of genes involved in neutrophil-mediated immunity, neutrophil activation involved in immune response, and neutrophil degranulation was reduced in COVID-19 compared to other-viral ARIs. However, neutrophil chemotaxis and neutrophil migration were increased in COVID-19 vs non-viral ARIs (Table. S3). Furthermore, the results of enriched MFs indicated that genes with CXCR3 chemokine receptor binding activity which play important role in recruiting pro-inflammatory cells such as neutrophils were upregulated in COVID-19. The regulation of the RIG-I signaling pathway, viral genome replication, and regulation of ribonuclease activity were incremented in the COVID-19 group compared to HC and non-viral groups. Moreover, the CCs related to the nucleus and cytosolic ribosome were overrepresented in the COVID-19 patients compared to HC.

According to the results, the NADH dehydrogenase complex which has an important role in the redox system by producing reactive oxygen species was increased in COVID-19 compared to non-viral ARIs. Furthermore, few genes involved in “protein polyubiquitination” were specifically upregulated in COVID-19 patients compared to other viral patients and the HC group (Fig. 4B and Additional file 4: Table S3).

Pathway enrichment analysis also confirmed that dysregulation of *CXCL10*, *CXCL11*, *CXCL9*, *DDX60*, *EPSTI1*, *IFI27*, *IFI44*, *IFIT2*, *IFIT3*, *ISG15*, *SAMD9L*, and *SAMHD1* genes in COVID-19 patients results in activation of interferon-alpha and gamma signaling pathways compared to HC and non-viral groups (Fig. 4C). However, apoptosis and p53 pathways were only enhanced in COVID- 19 patients compared to the non-viral group. The main dysregulated genes involved in these pathways were *GADD45A*, *IFITM3*, *IFNGR1*, and *IER3*.

On the other hand, the TNF-alpha signaling pathway was enriched in COVID-19 relative to other-viral ARIs. Moreover, some genes related to protein secretion and reactive oxygen species pathways like *MAPK1*, *RAB14*, *RAB22A*, *SOD1*, and *CAT* were dysregulated in COVID-19 versus other-viral diseases. All of these four sets of DEGs, BPs, MF, CCs, and enriched signaling pathways are accessible in Additional file 4: Table S3.

### Common and distinct gene signatures associated with COVID-19 in WB and NP samples

**Fig. 5:**
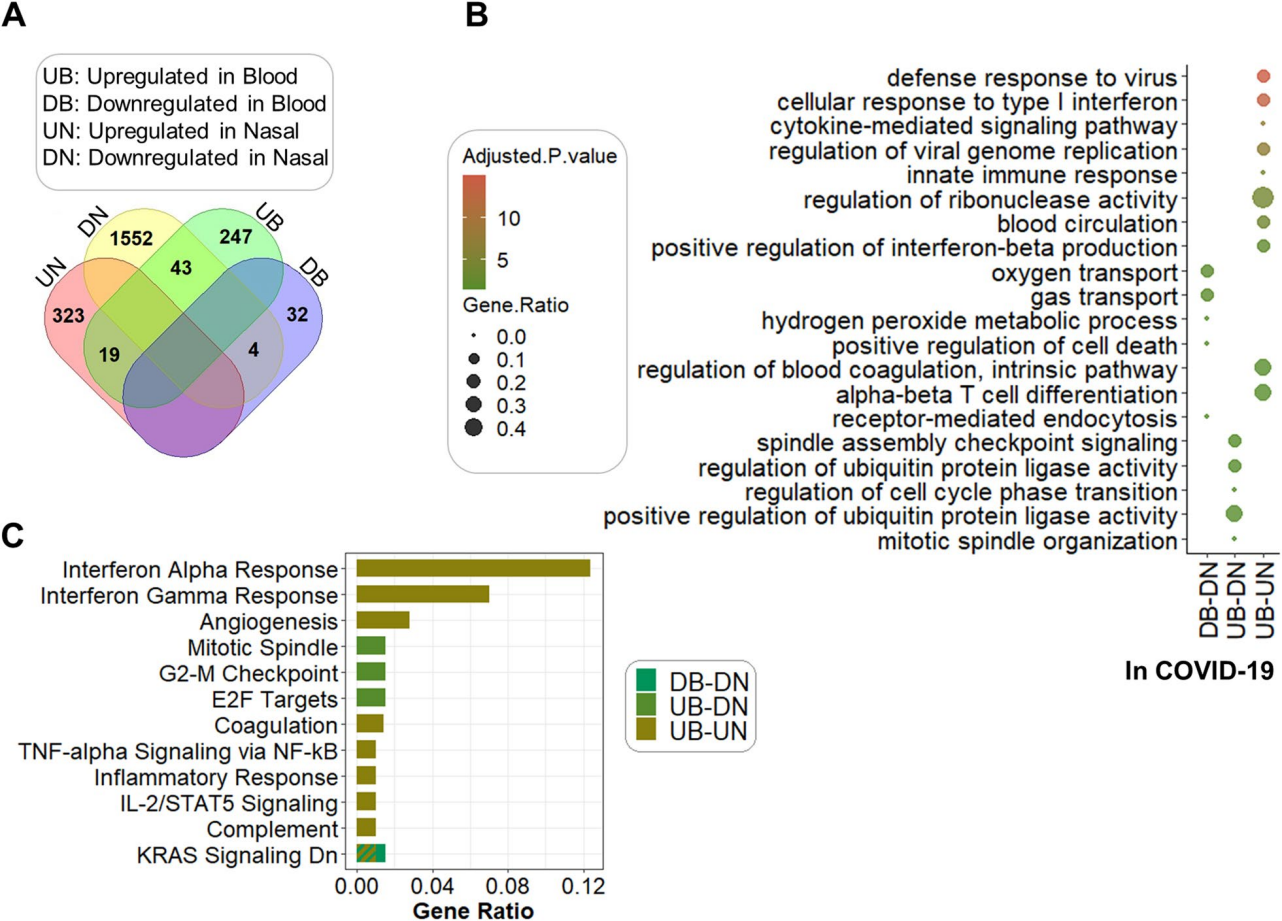
Analysis of common dysregulated genes in SARS-COV-2 -infected whole blood and nasopharyngeal samples in comparison with healthy controls: The Venn diagram to display the distribution of genes in four desired groups (*UB* upregulated genes in blood, *DB* downregulated genes in blood, *UN* upregulated genes in nasal, and *DN* downregulated genes in nasal) (**A**). Dot plot to show BPs according to common genes of each paired group. The size of the dots is proportional to the gene ratio in considering process and the color corresponds to the –log10 of the adjusted P-value. Selected top and not-redundant terms are visualized (**B**). Bar plot to depict hallmark gene set enrichment analysis. The size of the bars is proportional to the gene ratio in considering pathway and the color corresponds to paired groups whose common genes were studied. The “KRAS Signaling Dn” pathway was enriched in two groups (**C**). *BP* biological process

To investigate the overlapped molecular mechanisms involved in SARS-COV-2 host responses between WB and NP samples, common genes which were differentially expressed in WB and NP samples of COVID-19 patients compared to HC were determined. Using the Venn diagram, the common DEGs indicated in Fig. 5A were partitioned into three groups: upregulated in WB and upregulated in NP (UB-UN), downregulated in WB and downregulated in NP (DB-DN), and upregulated in WB and downregulated in NP (UB-DN).

The first group had 19 genes, and the next two groups contained 4 and 43 genes, respectively. The BPs related to innate immune response such as cellular response to IFNI, IFN-b production, defense response to virus, and cytokine signaling pathways, as well as alpha–beta T cell differentiation, were increased in both WB and NP groups, while the gas transportation, hydrogen peroxide metabolism, and cell death were reduced in both WB and NP groups (Fig. 5B). Also, consistent with the enriched “gas transportation” BP, MFs such as “heme binding” and “haptoglobin binding” were decreased in both WB and NP groups. Interestingly, the cell cycle and ubiquitin-protein ligase activity were enriched BPs in the UB-DN group. These findings were in line with functional enrichment analyses performed independently in the previous sections on WB and NP transcriptomes of patients with COVID-19. Most of the enriched BPs by *EnrichR* (Fig. 5B) were also validated by the *ToppGene* database (Additional file 5: Table S4).

Likewise, CCs validated by both *EnrichR* and *ToppGene* databases for DB-DN genes were related to endocytic vesicle lumen, cytosolic small ribosomal subunit, and small ribosomal subunit, which were in line with the previous enriched CCs obtained independently from SARS-COV-2 infected NP and WB transcriptome analyses. The common overexpressed genes of NP and WB samples, including *SERPING1*, *OLR1*, *IFITM3*, *CXCL10*, *IFI27*, *IFI44*, *IFI44L*, *IFIT3*, and *ISG15* were mostly members of complement, IL-2/STAT5, inflammatory response, interferon alpha, and interferon gamma responses, and TNF-alpha signaling pathways (Fig. 5C). Furthermore, we found that the *CDC20*, *PLK1*, and *BIRC5* genes lead to enriching E2F targets and the G2-M checkpoint pathway in the UB-DN group. Intriguingly, downregulation of KRAS signaling was observed in both UB-UN and DB-DN groups (Fig. 5C). The complete list of common DEGs in WB and NP samples of COVID-19 patients compared to the control group, significant BPs, MF, and CCs, and enriched signaling pathways based on *EnrichR* and *ToppGene* databases as well as their commonalities are available in Additional file 6: Table S5.

### Identification of high-performance diagnostic biomarker panels for COVID-19 in WB and NP samples

To determine the best diagnostic biomarker panel, in the first phase, the DEGs associated with train sets of WB and NP samples were applied as inputs to the LASSO regression method for feature selection. We selected 80% of each group of WB and NP samples as train sets using a stratified random sampling method. The genes with an absolute LASSO coefficient more than 0.1 OR the genes with a non-zero LASSO coefficient and an absolute value of logFC more than 1.3 were selected (described in Table 2), and used in the RF classifier. According to the mentioned criteria, 22 and 23 markers related to WB and NP datasets were detected and almost all of them have previously been implicated in COVID-19 or other viral or inflammatory illnesses.

**Table 2:** The selected features based on the criteria* in the train sets of WB and NP samples

NP					WB				
Gene	L.Coef	Abs.L.Coef	logFC	Abs.logFC	Gene	L.Coef	Abs.L.Coef	logFC	Abs.logFC
IFI6	− 0.28491	0.284913	1.613021	1.613021	SLC24A5	− 1.00469	1.004693	− 2.03768	2.037676
IFI44L	− 0.27956	0.279561	1.970857	1.970857	SLC45A2	0.706125	0.706125	1.208076	1.208076
SIGLEC1	− 0.27801	0.278012	1.332828	1.332828	C1QC	0.416476	0.416476	1.196718	1.196718
NUCB1	0.226323	0.226323	− 1.00591	1.005905	NMNAT2	0.403256	0.403256	1.065142	1.065142
XAF1	− 0.21556	0.215562	1.081608	1.081608	LGSN	0.313768	0.313768	1.034986	1.034986
TMED9	0.201803	0.201803	− 1.50563	1.505627	HIST2H4A	0.306524	0.306524	2.531967	2.531967
SAMHD1	0.168243	0.168243	− 1.00134	1.001343	INSC	0.300362	0.300362	1.489929	1.489929
SDC1	− 0.15729	0.157293	− 1.00398	1.003981	GOLGA8M	0.288115	0.288115	1.033698	1.033698
TIMM13	0.154103	0.154103	− 1.1873	1.187303	CDCA5	0.209252	0.209252	1.05616	1.05616
IL1R2	0.14483	0.14483	− 1.37001	1.370005	NOS1AP	0.194112	0.194112	1.137223	1.137223
CXCL11	0.122537	0.122537	1.340965	1.340965	BEGAIN	0.190819	0.190819	1.167257	1.167257
LAMB3	0.092874	0.092874	− 1.46286	1.462858	OTOF	0.171533	0.171533	1.280815	1.280815
TMA7	0.081283	0.081283	− 1.315	1.314995	UGT2B11	− 0.15846	0.158463	− 1.48498	1.484976
ADIRF	0.069208	0.069208	− 1.30839	1.308395	GSTM1	− 0.15549	0.155486	− 1.43369	1.433691
BBS10	0.05455	0.05455	− 1.40761	1.407608	OR10G2	− 0.13769	0.137689	− 1.95528	1.95528
OR1I1	0.050515	0.050515	− 1.4272	1.427201	TRIP13	0.115733	0.115733	1.028476	1.028476
MIF	0.031292	0.031292	− 1.80266	1.802656	CCDC27	− 0.10699	0.106987	− 1.99933	1.999328
CXCL10	0.029183	0.029183	1.5632	1.5632	ABCC11	0.08954	0.08954	1.31761	1.31761
C19orf33	0.015395	0.015395	− 1.33282	1.33282	EIF1AY	− 0.08357	0.083573	− 1.76969	1.769694
COPA	0.014771	0.014771	− 1.31686	1.316856	OR2A42	− 0.0225	0.022504	− 2.24545	2.245455
ADAM17	0.012107	0.012107	− 1.36326	1.363259	GTF2H2C	0.01273	0.01273	1.429336	1.429336
TCTEX1D4	0.006927	0.006927	− 1.52527	1.52527	SCN5A	0.007577	0.007577	1.405947	1.405947
IFIT2	0.005005	0.005005	1.308344	1.308344					

*WB* whole blood, *NP* nasopharyngeal

*Absolute LASSO coefficient more than 0.1 OR the non-zero LASSO coefficient and the absolute value of logFC more than 1.3

**Fig. 6:**
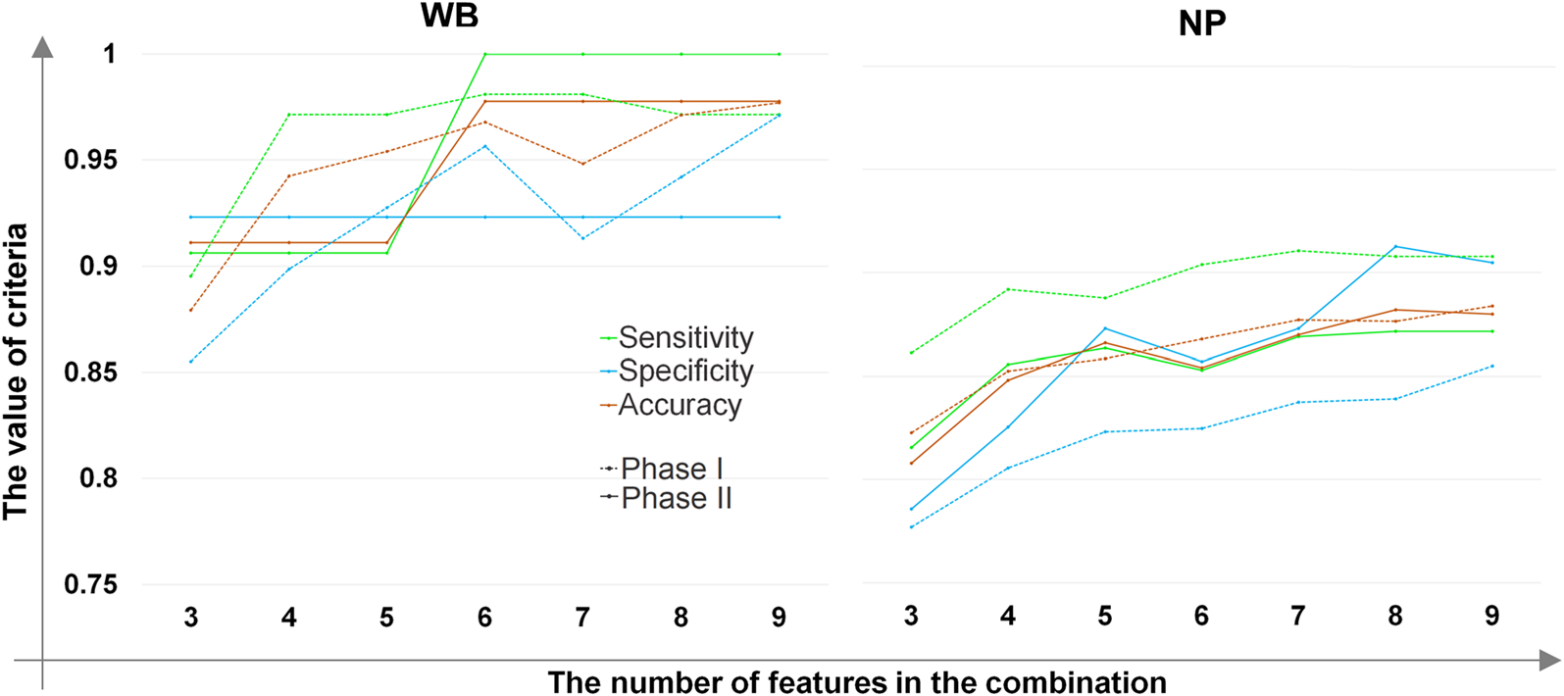
The criteria of classifiers: The Line plots to indicate the value of the sensitivity, specificity, and accuracy of the classifiers for whole blood (WB) and nasopharyngeal (NP) samples in the first and second phases based on the number of features

**Fig. 7:**
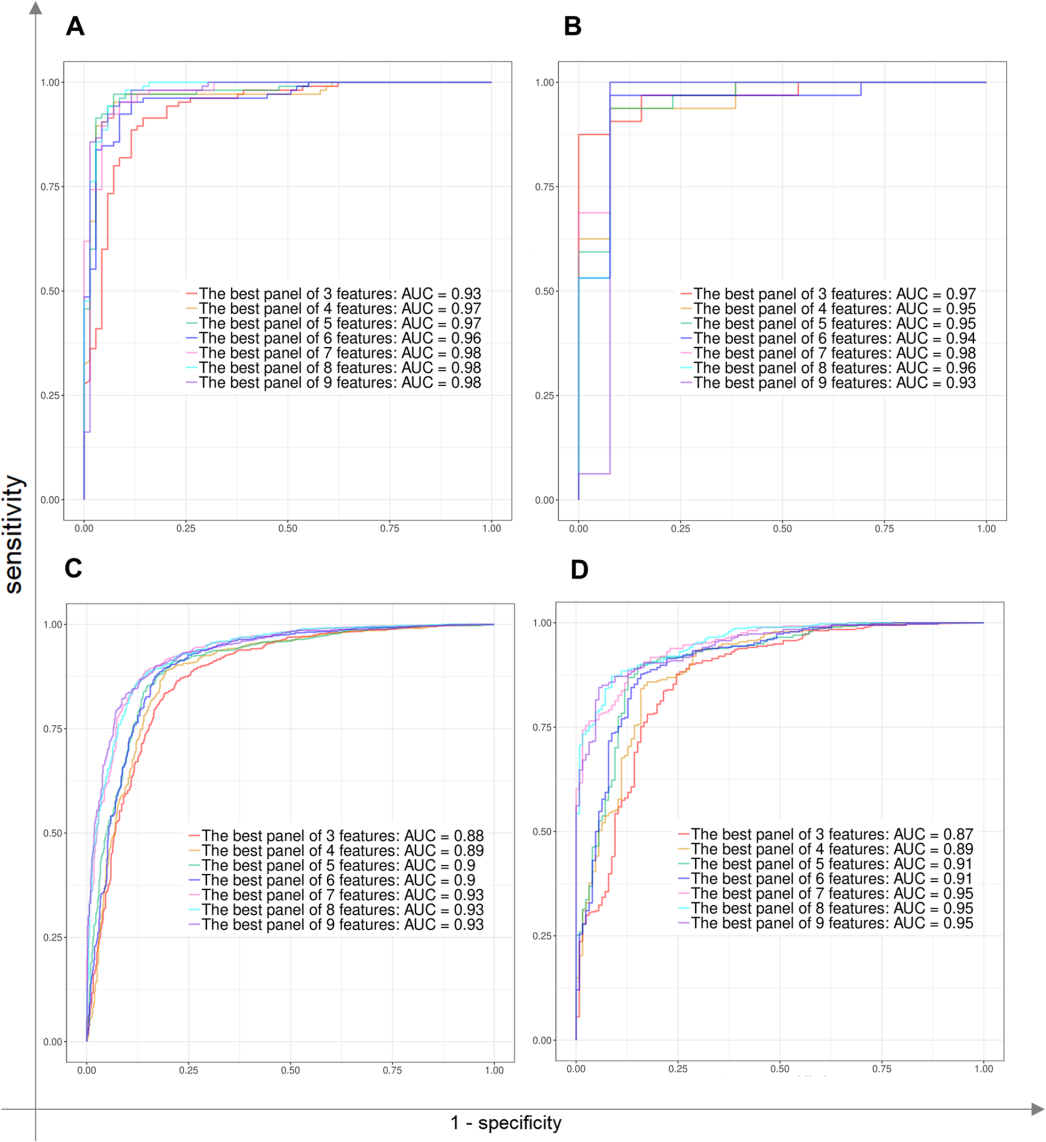
The ROC curves: These ROC curves illustrate the sensitivity, 1-specificity, and AUC associated to phase I (**A** and **C**) and phase II (**B** and **D**) for whole blood and nasopharyngeal samples among the top 3 to 9 features, respectively

The RF classification was implemented on all combinations of 3 to 9 features based on 5/tenfold cross validation in WB/NP training sets. The biomarker panels were designated to enter the next phase based on different values of Accuracy. Considering that the number of combinations for 3 to 9 features varied from 4620 to 4,476,780, in order to keep the calculation cost-effective, it was desirable that as few biomarker panels as possible be included in subsequent evaluations. Consequently, the biomarker panels with the accuracy of 75% for 3-marker panels to 85% for 9-marker panels were selected in WB datasets to enter the next phase. These criteria in the NP dataset were 80% for 3-marker panels to 85% for 9-marker panels, as well. 5272 and 1259 combinations for WB and NP samples were selected to be applied in the second phase. In the next phase, the RF classifier was applied on independent test sets to validate the best selected biomarker panels. The employed methodology gained the final panels based on more data than any of the previous studies and also was validated twice. Therefore, these biomarker combinations could be more generalized, robust, and powerful. The parameters related to the first and second phases for the best combinations of markers were illustrated in Additional file 6: Table S5 and Table 3. The best final 3- to 9-marker panels to diagnose COVID-19 in WB samples had an accuracy of 88% to 98% in the first phase and 91% to 98% in the second phase. Similarly, the best final 3- to 9-marker panels for classifying NP samples had an accuracy of 82% to 88% in the first phase and 80% to 88% in the second one. Correspondingly, line plots of the sensitivity, specificity, and accuracy as well as ROC curves obtained from two phases for NP and WB samples were presented in Figs. 6 and 7.

**Table 3:** The criteria obtained for WB and NP samples in the first and second phases by the RF classifier based on LASSOfeatures

Tissue	The first phase based on the k-fold CV on the train set			The second phase based on train and test sets			Number of features	Genes
	Sensitivity_cv	Specificity_cv	Accuracy_cv	Sensitivity	Specificity	Accuracy		
WB	0.895238095	0.855072464	0.879310345	0.90625	0.923076923	0.911111111	3	CCDC27, CDCA5, EIF1AY
	0.971428571	0.898550725	0.942528736	0.90625	0.923076923	0.911111111	4	CCDC27, HIST2H4A, NOS1AP, TRIP13
	0.971428571	0.927536232	0.954022989	0.90625	0.923076923	0.911111111	5	CCDC27, HIST2H4A, LGSN, NOS1AP, TRIP13
	0.980952381	0.956521739	0.967816092	1	0.923076923	0.977777778	6*	C1QC, CCDC27, HIST2H4A, INSC, OTOF, SLC24A5
	0.980952381	0.956521739	0.967816092	1	0.923076923	0.977777778	6	CCDC27, CDCA5, INSC, NMNAT2, OR2A42, SLC24A5
	0.980952381	0.913043478	0.948275862	1	0.923076923	0.977777778	7	ABCC11, CCDC27, INSC, NMNAT2, OTOF, SLC24A5, TRIP13
	0.971428571	0.942028986	0.971264368	1	0.923076923	0.977777778	8	ABCC11, CCDC27, CDCA5, GTF2H2C, INSC, OTOF, SLC24A5, SLC45A2
	0.971428571	0.971014493	0.977011494	1	0.923076923	0.977777778	9*	CCDC27, CDCA5, EIF1AY, GOLGA8M, GTF2H2C, INSC, NMNAT2, OTOF, SLC24A5
	0.971428571	0.971014493	0.977011494	1	0.923076923	0.977777778	9	CCDC27, GSTM1, GTF2H2C, INSC, NMNAT2, NOS1AP, OTOF, SLC24A5, TRIP13
NP	0.861148198	0.777070064	0.822803195	0.815508021	0.785714286	0.808	3	IFI6, MIF, NUCB1
	0.891855808	0.805732484	0.852578068	0.855614973	0.825396825	0.848	4	IFI6, LAMB3, MIF, NUCB1
	0.887850467	0.823248408	0.8583878	0.863636364	0.873015873	0.866	5	IFI44L, IFI6, IL1R2, MIF, NUCB1
	0.903871829	0.824840764	0.867828613	0.852941176	0.857142857	0.854	6	ADIRF, IFI6, IL1R2, MIF, NUCB1, TMA7
	0.910547397	0.837579618	0.877269426	0.868983957	0.873015873	0.87	7	CXCL10, IFI6, MIF, NUCB1, SAMHD1, SIGLEC1, TMED9
	0.90787717	0.839171975	0.87654321	0.871657754	0.912698413	0.882	8	COPA, CXCL11, IFI6, MIF, NUCB1, SAMHD1, SIGLEC1, TMED9
	0.90787717	0.855095541	0.883805374	0.871657754	0.904761905	0.88	9	CXCL11, IFI6, IFIT2, LAMB3, MIF, NUCB1, SAMHD1, TMA7, XAF1

*WB* whole blood, *NP* nasopharyngeal

*Selected to depict ROC curves

The best biomarker panels obtained from the LASSO method, as subsets of DEGs, were part of CMS achieved by gene set enrichment and GO analyses of WB and NP transcriptomes. method. Functional enrichment analysis based on *EnrichR* and *ToppGene* databases showed that WB-based diagnostic markers were mostly involved in cell cycle-related BPs and pathways. Besides, in agreement with previous results, the “hemoglobin biosynthetic process” and “hemoglobin metabolic process” were decreased MFs, indicating the important role of candidate biomarkers in COVID-19 pathogenesis. Functional annotation of NP biomarkers indicated that the MFs of “antiviral innate immune response,” “cellular response to cytokine stimulus,” and “positive regulation of immune system process” were enhanced. They significantly contributed to interferon signaling pathways (Additional file 7: Table S6). In the next step, we investigated whether 23 common DEGs in WB and NP samples of COVID-19 patients compared to the control group could be used as robust features to classify COVID-19 from non-COVID cases using the RF algorithm. The total number of combinations with 3 to 9-markers related to 23 common DEGs was 1,697,883. we selected the first 100 top panels with the highest accuracy and sensitivity for each class (Additional file 8: Table S7).

We then compared the results of the LASSO feature-based prediction model and the common WB/NP feature-based prediction model (an RF-based generic prediction model) (Table 4). The genes IFI44L, IFI6, and CXCL10 were observed in the best NP-based diagnostic panels of both prediction models. Likewise, GTF2H2C as a novel gene related to SARS-COV-2 infection was presented in the best WB-based diagnostic panels of the LASSO- and common feature-based prediction models.

**Table 4:** The comparison of criteria related to the best panels of the LASSO feature-based prediction model and RF-based generic prediction model

Tissue	Number of features	The best LASSO-based panels based on train and test sets			The best common-based panels based on train and test sets			Difference of accuracies
		Sensitivity	Specificity	Accuracy	Sensitivity	Specificity	Accuracy	
WB	3	0.906	0.923	0.911	0.938	0.769	0.889	0.022
	4	0.906	0.923	0.911	0.969	0.692	0.889	0.022
	5	0.906	0.923	0.911	0.969	0.769	0.911	0.000
	6	1	0.923	0.978	0.938	0.846	0.911	0.067
	7	1	0.923	0.978	0.938	0.846	0.911	0.067
	8	1	0.923	0.978	0.938	0.846	0.911	0.067
	9	1	0.923	0.978	0.906	0.846	0.889	0.089
NP	3	0.816	0.786	0.808	0.743	0.802	0.758	0.050
	4	0.856	0.825	0.848	0.810	0.810	0.810	0.038
	5	0.864	0.873	0.866	0.826	0.841	0.830	0.036
	6	0.853	0.857	0.854	0.829	0.865	0.838	0.016
	7	0.869	0.873	0.870	0.832	0.881	0.844	0.026
	8	0.872	0.913	0.882	0.850	0.841	0.848	0.034
	9	0.872	0.905	0.880	0.850	0.857	0.852	0.028

*WB* whole blood, *NP* nasopharyngeal

Our findings indicated that the accuracy of the best panels in the LASSO feature-based prediction model was higher than that in the RF-based generic prediction model. In addition, as represented in Additional file 6: Table S5 and Additional file 8: Table S7, LASSO-based panels have higher performance in comparison to biomarker panels which were achieved using common WB/NP DEGs.

## Discussion

Understanding the pathogenesis of COVID-19 plays a key role in drug design and treatment. Besides, identifying the host responses in different tissues can give us a more comprehensive conception.

In the present study, we implemented a meta-analysis and integrated 9 public datasets from different populations to improve individual study-specific biases. Using this approach, we notably increased the sample size and decreased the heterogeneity of the samples compared to previous studies. we applied GO and enrichment analyses to scrutinize more pathophysiological aspects of this novel pandemic virus compared to other ARIs and physiological conditions and developed the diagnostic panels based on host WB and NP transcriptomes using the ML methods.

The results revealed the distinct gene expression patterns of WB and NP samples in patients with COVID-19 versus other individuals. In agreement with previous studies (Thair et al. 2020a; Aschenbrenner et al. 2020), WB transcriptomics followed by the analysis of immune cell type proportion showed an increase in the number and activity of neutrophils in COVID-19 patients compared to healthy individuals.

T cells have been found to play a key role in viral infections and immunological homeostasis. The observed decline in CD4^+^ and CD8^+^ T cells could be attributed to SARS-COV-2 replication and dissemination, and the associated immunopathology damage (Huang et al. 2021). Despite the reduction of CD4^+^ and CD8^+^ T lymphocyte subsets in COVID-19-infected patients, our deconvolution analysis demonstrated that the Treg population was highly increased. These findings imply that Tregs may exert a negative effect on COVID-19 patients via inhibiting antiviral T-cell responses during the infection (Galván-Peña et al. 2021). In particular, our research showed that naïve B cells and plasma cells were highly increased in COVID-19 whose antibody production may prevent COVID-19 patients from deteriorating) (Huang et al. 2021; Wu et al. 2020). Transcriptome analysis revealed that the oxygen transport process was decremented in WB samples of COVID-19 patients, corresponding to the clinical features of hypoxia. Hypoxia not only plays an essential role in inflammation by increasing the release of pro-inflammatory cytokines but may also affect viral replication (Tavassolifar et al. 2020, 2021; Zhou et al. 2021; Maras et al. 2021), an enriched BP which was also observed in this study.

Impaired regulation of host antiviral immune responses, such as IFN pathway activation and chemokine production, is part of the characteristics of viral infections (Salazar-Mather and Hokeness 2006; McNab et al. 2015). In line with previous studies (Kim and Shin 2021; Hadjadj et al. 2020), the IFN-mediated antiviral signature was obtained in the WB and NP samples of SARS-COV-2 infected patients as a crucial immune system response. Serum IFN-I levels in COVID-19 patients are higher in comparison to HC, proposing a beneficial effect of enhanced IFN-I in the blood for killing SARS-COV-2 (Huang et al. 2021). However, IFN-mediated antiviral pathways, innate immune responses, and response to virus pathways were less activated in NP samples infected by SARS-COV-2 compared to other respiratory viruses. Further, it has been mentioned that specific expression patterns of IFN pathway activation in SARS-COV-2 differ from those found in other respiratory viruses (Ng et al. 2021). A specific immune response is necessary during the incubation and non-severe phases to eradicate the virus and prevent disease progression to severe stages. SARS-COV-2 replicates during the incubation period and severely damages the targeted tissues, particularly in organs with high ACE2 levels, by impairing the protective immune response (Shi et al. 2020). Consequently, the lower innate immune response and inflammation compared to other viruses during the incubation period may lead to enhance viral replication and propagation and subsequent enormous destruction of the affected tissues. In general, transcriptome analysis of WB and NP samples illustrated that the expression pattern of some genes is altered in SARS-COV-2 compared to HCs. Some of them like genes involved in the cell cycle showed an opposite expression pattern in SARS-COV-2-infected NP and WB samples which means that the pathogenesis of COVID-19 is body-site-specific. Albeit, due to the common DEGs in WB and NP samples of COVID-19 patients versus HC, such as those involved in the INF and cytokine pathways, we showed that SARS-COV-2 could also induce a global and systematic host response.

RT-PCR, as the gold standard method of SARS-COV-2 detection, has clinical sensitivity ranging from 66 to 80% (Nag et al. 2020). Hence, host transcriptional biomarkers could be employed alone or combined with molecular detection of SARS-COV-2 to reduce false-negative and false-positive results, such as those caused by insufficient viral load or cross-contamination (Mick et al. 2020). We attempted to address this problem by using two meta-datasets from WB and NP samples to develop COVID-19 diagnostic biomarker panels that are precise and clinically practical and can overcome the constraints of direct viral genetic material detection. The differential gene expression analysis followed by the LASSO method led to the detection of 22 and 23 markers for WB and NP, respectively. The high-performance 3 to 9-gene sets of WB samples were determined from 22 features. We also applied 23 common WB/NP DEGs as input features to construct RF-based prediction models and observed that the accuracy, sensitivity, and specificity of these models were less than LASSO feature-based prediction models. Intriguingly, both prediction models included some common markers, such as the GTF2H2C gene, which was found in the best WB-based diagnostic panels in both prediction models.

Most of the 22 features of WB which were achieved by the LASSO method introduced as key factors in the pathogenesis of SARS-COV-2 or other viral infections in previous studies. For instance, ABCC11 is one of the ABC transporter genes whose genetic polymorphism has been associated with SARS-COV-2 infection (Yamamoto et al. 2021). Likewise, transcription of complement genes such as C1QC has been reported to be induced in response to SARS-COV-2, while higher expression has been observed in the lung relative to blood (Daamen et al. 2021; Lazara et al. 2021). A protein–protein interaction analysis also showed that CDCA5, a gene involved in the cell cycle, could be one of the significant characteristics of SARS-COV-2 infection (Mo et al. 2021). The GSTM1 gene, another introduced biomarker increases the risk of various oxidative stress-related multifactorial disorders, it might therefore play a role in susceptibility to SARS-COV-2 infection, as well (Abbas et al. 2021). Although the association of some biomarkers such as SCN5A and BEGAIN with SARS-COV-2 infection has been previously reported (Guidicessai et al. 2020; Thair et al. 2020b), their specific function in SARS-COV-2 pathogenesis needs to be experimentally clarified. Furthermore, the importance of some features like GOLGA8M and SLC24A5 has been stated in the other viral infections or autoimmune diseases. GOLGA8M, which encodes Golgin A8 family member M, contributes to the development of HBV-related HCC (Jiang et al. 2020). The researchers have shown that the transport protein SLC24A5 induces a significantly higher frequency of CD8 T cell activation in an autoimmune disease like Alopecia areata (Wang et al. 2019).

The NP-based diagnostic biomarker panels that could discriminate COVID-19 patients from non-COVID individuals were obtained from 23 features. Among them, the overexpression of pro-inflammatory chemokines and cytokines is a key hallmark of SARS-COV-2-induced pulmonary complications, which contribute to a cytokine storm (Callahan et al. 2021). The gene CXCL10 as a pro-inflammatory chemokine, which was presented in the best panels of both prediction models, could be one of the main mediators involved in the SARS-COV-2-related cytokine storm. The expression level of CXCL10 has shown a strongly significant positive correlation with viral load and progression of COVID-19, thus it could be also used as a biomarker for COVID-19 acute respiratory distress syndrome (ARDS) patients (Oliviero et al. 2021; Hemida et al. 2010). Furthermore, the significant overexpression of CXCL11 transcripts has been demonstrated in patients with mild to severe disease, which may be related to different T cell responses in COVID-19 patients (Yang et al. 2020). Macrophage migration inhibitory factor (MIF), another biomarker candidate, has an important role in the inflammatory response to SARS-COV-2 infection by inducing the pulmonary inflammatory cytokines (Dheir et al. 2021). In addition, it has been observed that the level of MIF is higher in severe patients with COVID-19 (Aksakal et al. 2021). Therefore, MIF was also identified as a biomarker for determining the patients with COVID-19 ARDS (Bleilevens et al. 2021). We also identified that the interferon-stimulated genes (ISGs) such as *IFIT2*, *IFI6*, and I*FI44L* were upregulated in COVID-19 patients. Studies have shown that *IFIT2* and *IFI44L* are strongly induced in bronchoalveolar lavage (BAL) cells and peripheral blood mononuclear cells (PBMCs) of COVID-19 patients, leading to enhance antiviral and immune modulatory functions (Zhu et al. 2020; Shaath et al. 2020). Transcriptome analysis of PBMCs also highlighted the potential role of IFI6 in responses to SARS-COV-2 in comparison to other respiratory viruses (Shaath et al. 2020; Qi et al. 2021). According to our findings, IL1R2 is a downregulated NP marker for COVID-19 patients. *IL1R2*, known as anti-inflammatory cytokines, is highly expressed in monocyte-macrophages from BALF and follicular regulatory T (TFR) cells; however, TFR cells have been reported to be significantly lower in hospitalized COVID-19 patients (Meckiff et al. 2020; Xu et al. 2020). LAMB3 which is present in anchoring junctions of epithelial cells was among NP-based diagnostic biomarkers. The human papillomavirus penetrates the epithelial barrier by inducing some changes in the LAMB3 of anchoring junctions (Dong et al. 2020). Evidence has shown that blockers of anchoring junction proteins such as LAMB3 could prevent COVID-19 infection (Doehn et al. 2021). SAMHD1 is the molecule that controls the cellular deoxyribonucleoside triphosphates (dNTP) pool and has an inhibitory effect on HIV-1 replication by reducing the concentration of intracellular dNTP pools (Monit et al. 2019; Buffone et al. 2019). It has been suggested that SAMHD1 may be associated with neurological complications of COVID-19 (Khan and Sergi 2020). XIAP-associated factor 1 (XAF1), as a novel binding ligand of XIAP, can reverse XIAP's anti-apoptotic activity (Gao et al. 2021). Zhu et al. reported that the expression of XAF1 in T, B, NK, and DC cells from patients with COVID-19 and influenza was upregulated, thus it may increase apoptosis in T-cells of COVID-19 patients (Zhu et al. 2020).

The best final host response-based markers were acquired using a 2-phase ML approach which employed k-fold cross validation on discovery sets (80% of the population) in the first step by all 3 to 9 combinations of selected features. The best results were then validated on independent test datasets (20% of the population), as well. Data integration from several different laboratories followed by a 2-phase ML method made the final host response-based biomarker panels more powerful, robust, and generalized. The optimal WB- and NP-based diagnostic panels which have the minimum number of markers with maximum accuracy included 6 and 8 markers with an accuracy of 97% and 88% in both two phases.

## Conclusion

Distinct transcriptome profiles of different SARS-COV-2-infected tissues relative to HCs and other pathophysiological conditions shed the light on the necessity of SARS-COV-2-specific drug design. Comparison of gene expression profiles of WB and NP samples with HCs demonstrated that expression of some genes is exclusively altered in WB or NP samples. Intriguingly, some of them like genes involved in the cell cycle showed a remarkably opposite expression pattern which means that the pathogenesis of COVID-19 may be body-site-specific. On the other hand, SARS-COV-2 induces a global and systematic host response according to common gene signatures in WB and NP e.g. the genes involved in INF and cytokine pathways, demonstrating the disease's complexity, as well. We introduced and validated host-response-based diagnostic biomarkers using ML methods which could be applied as a complementary tool to diagnose the COVID-19 infection from non-COVID cases.

## Supplementary Information

**Additional file 1: Fig. S1.** PCA plots for batch effect removal using ComBat-seq in Whole Blood samples: The PCA plots show the samples of RNA-seq datasets before (A) and after (B) batch effect removal. **Fig. S2.** PCA plots for batch effect removal using ComBat-seq in nasopharyngeal samples: The PCA plots show the samples of RNA-seq datasets before (A) and after (B) batch effect removal.

**Additional file 2: Table S1.** Differentially expressed genes of whole blood (WB) when COVID-19 patients compared with healthy control (HC) individuals; significant Biological Processes (BPs), molecular functions (MFs), and cellular components (CCs) categories obtained from *EnrichR* and *ToppGene* databases; Significant hallmark pathways and enriched pathways acquired by *EnrichR* and *ToppGene* databases; The common results of GO-terms and enriched pathways between *EnrichR* and *ToppGene* databases.

**Additional file 3: Table S2.** The results of independent T-test between different immune cell types of COVID-19 and healthy control groups based on the Cibersort.

**Additional file 4: Table S3.** Differentially expressed genes, significant biological processes (BPs) molecular functions (MFs), and cellular components (CCs) categories obtained from *EnrichR* and *ToppGene* databases; Significant hallmark pathways and enriched pathways acquired by *EnrichR* and *ToppGene* databases; The common results of GO-terms and enriched pathways between *EnrichR* and *ToppGene* databases when nasopharyngeal transcriptome of COVID-19 compared with that of healthy controls/non-viral acute respiratory illnesses (ARIs)/other viral ARIs.

**Additional file 5: Table S4.** Common differentially expressed genes between SARS-COV-2 infected whole blood and nasopharyngeal samples when compared with healthy controls; Significant biological processes (BPs) molecular functions (MFs), and cellular components (CCs) categories obtained from *EnrichR* and *ToppGene* databases; Significant hallmark pathways and enriched pathways acquired by *EnrichR* and *ToppGene* databases; The common results of GO-terms and enriched pathways between *EnrichR* and *ToppGene* databases.

**Additional file 6: Table S5.** The statistical criteria for the best combinations of the features obtained from the LASSO method in the first and second phases based on Random Forest (RF) classification among whole blood and nasopharyngeal data.

**Additional file 7: Table S6. **The significant GO terms and enriched pathways for LASSO features of WB and NP samples.

**Additional file 8: Table S7. **The statistical criteria for top 100 panels (3 to 9-diagnostic markers) obtained from the common WB/NP DEGs based on the train and test sets in WB and NP samples.

## Data Availability

All data generated in the study are included in the present article and additonal files.

## Abbreviations

WB: Whole-blood
NP: Nasopharyngeal
ARIs: Acute respiratory illnesses
HCs: Healthy controls
ML: Machine learning
SARS-COV-2: Severe Acute Respiratory Syndrome Coronavirus 2
COVID-19: Coronavirus disease 2019
TNF: Tumor necrosis factor
RT-PCR: Reverse transcription-polymerase chain reaction
CPN: Cross-platform normalization
GEO: Gene expression omnibus
CMS: COVID-19 meta-signature’
GSE: Genomic spatial event
DEGs: Differentially expressed genes
eBayes: Empirical Bayes
FDR: False discovery rate
BP: Biological processes
CPM: Counts per million
GEPs: Gene expression profiles
LASSO: Least absolute shrinkage and selection operator
RF: Random forest
ROC: Receiver operating characteristic
PCA: Principal components analysis

## References

[CR1] Abbas M, Verma S, Verma S (2021). Association of GSTM1 and GSTT1 gene polymorphisms with COVID-19 susceptibility and its outcome. J Med Virol.

[CR2] Ahmed FF, Reza MS, Sarker MS (2022). Identification of host transcriptome-guided repurposable drugs for SARS-CoV-1 infections and their validation with SARS-COV-2 infections by using the integrated bioinformatics approaches. PLoS ONE.

[CR3] Aksakal A, Kerget B, Kerget F, Aşkın S (2021). Evaluation of the relationship between macrophage migration inhibitory factor level and clinical course in patients with COVID-19 pneumonia. J Med Virol.

[CR4] Andres-Terre M, McGuire HM, Pouliot Y (2015). Integrated, multi-cohort analysis identifies conserved transcriptional signatures across multiple respiratory viruses. Immunity.

[CR5] Aschenbrenner AC, Mouktaroudi M, Krämer B (2020). Disease severity-specific neutrophil signatures in blood transcriptomes stratify COVID-19 patients. Genome Med.

[CR6] Benjamini Y, Hochberg Y (1995). Controlling the false discovery rate: a practical and powerful approach to multiple testing. J R Stat Soc Ser B.

[CR7] Bleilevens C, Soppert J, Hoffmann A (2021). Macrophage migration inhibitory factor (Mif) plasma concentration in critically ill covid-19 patients: a prospective observational study. Diagnostics.

[CR8] Breiman L (2001). Random forests. Mach Learn.

[CR9] Buffone C, Kutzner J, Opp S (2019). The ability of SAMHD1 to block HIV-1 but not SIV requires expression of MxB. Virology.

[CR10] Callahan V, Hawks S, Crawford MA (2021). The pro-inflammatory chemokines cxcl9, cxcl10 and cxcl11 are upregulated following SARS-COV-2 infection in an akt-dependent manner. Viruses.

[CR11] Chen J, Bardes EE, Aronow BJ, Jegga AG (2009). ToppGene Suite for gene list enrichment analysis and candidate gene prioritization. Nucleic Acids Res.

[CR12] Chen B, Khodadoust MS, Liu CL (2018). Profiling tumor infiltrating immune cells with CIBERSORT. Methods Mol Biol.

[CR13] Chen G, Wu D, Guo W (2020). Clinical and immunological features of severe and moderate coronavirus disease 2019. J Clin Invest.

[CR14] Daamen AR, Bachali P, Owen KA (2021). Comprehensive transcriptomic analysis of COVID-19 blood, lung, and airway. Sci Rep.

[CR15] Danford T, Rolfe A, Gifford D (2008). GSE: a comprehensive database system for the representation, retrieval, and analysis of microarray data. Pacific Symp Biocomput 2008. PSB.

[CR16] Dheir H, Yaylaci S, Sipahi S (2021). Does macrophage migration inhibitory factor predict the prognosis of COVID-19 disease?. J Infect Dev Ctries.

[CR17] Doehn JM, Tabeling C, Biesen R (2021). CD169/SIGLEC1 is expressed on circulating monocytes in COVID-19 and expression levels are associated with disease severity. Infection.

[CR18] Dong D, Xie W, Liu M (2020). Alteration of cell junctions during viral infection. Thorac Cancer.

[CR19] Galván-Peña S, Leon J, Chowdhary K (2021). Profound Treg perturbations correlate with COVID-19 severity. Proc Natl Acad Sci U S A.

[CR20] Gao X, Liu Y, Zou S (2021). Genome-wide screening of SARS-COV-2 infection-related genes based on the blood leukocytes sequencing data set of patients with COVID-19. J Med Virol.

[CR21] Giudicessi JR, Roden DM, Wilde AAM, Ackerman MJ (2020). Genetic susceptibility for COVID-19-associated sudden cardiac death in African Americans. Heart Rhythm.

[CR22] Hadjadj J, Yatim N, Barnabei L (2020). Impaired type I interferon activity and inflammatory responses in severe COVID-19 patients. Science (80-)..

[CR23] Hamid JS, Hu P, Roslin NM (2009). Data integration in genetics and genomics: methods and challenges. Hum Genomics Proteomics.

[CR24] Hemida MG, Ye X, Thair S, Yang D (2010). Exploiting the therapeutic potential of microRNAs in viral diseases: expectations and limitations. Mol Diagnosis Ther.

[CR25] Huang L, Shi Y, Gong B (2021). Dynamic blood single-cell immune responses in patients with COVID-19. Signal Transduct Target Ther.

[CR26] Irigoyen A, Jimenez-Luna C, Benavides M (2018). Integrative multi-platform meta-analysis of gene expression profiles in pancreatic ductal adenocarcinoma patients for identifying novel diagnostic biomarkers. PLoS ONE.

[CR27] Jiang D, Deng J, Dong C (2020). Knowledge-based analyses reveal new candidate genes associated with risk of hepatitis B virus related hepatocellular carcinoma. BMC Cancer.

[CR28] Khan A, Sergi C (2020). SAMHD1 as the potential link between SARS-COV-2 infection and neurological complications. Front Neurol.

[CR29] Kim YM, Shin EC (2021). Type I and III interferon responses in SARS-COV-2 infection. Exp Mol Med.

[CR30] Kuleshov MV, Jones MR, Rouillard AD (2016). Enrichr: a comprehensive gene set enrichment analysis web server 2016 update. Nucleic Acids Res.

[CR31] L’Heureux A, Grolinger K, Elyamany HF, Capretz MAM (2017). Machine learning with Big Data: challenges and approaches. IEEE Access..

[CR32] Larsen MJ, Thomassen M, Tan Q (2014). Microarray-based RNA profiling of breast cancer: batch effect removal improves cross-platform consistency. Biomed Res Int.

[CR33] Lazara S-L, Amamura TA, da Silva TF (2021). A double edged-sword—the complement system during SARS-COV-2 infection. Life Sci.

[CR34] Liao M (2020). Single-cell landscape of bronchoalveolar immune cells in patients with COVID-19. Nat Med.

[CR35] Lieberman NAP, Peddu V, Xie H (2020). In vivo antiviral host transcriptional response to SARS-COV-2 by viral load, sex, and age. PLoS Biol.

[CR36] Maleknia S, Salehi Z, Rezaei Tabar V (2020). An integrative Bayesian network approach to highlight key drivers in systemic lupus erythematosus. Arthritis Res Ther.

[CR37] Maras JS, Sharma S, Bhat A (2021). Multi-omics analysis of respiratory specimen characterizes baseline molecular determinants associated with SARS-COV-2 outcome. iScience.

[CR38] McNab F, Mayer-Barber K, Sher A (2015). Type I interferons in infectious disease. Nat Rev Immunol.

[CR39] Meckiff BJ, Ramírez-Suástegui C, Fajardo V (2020). Imbalance of regulatory and cytotoxic SARS-COV-2-reactive CD4+ T cells in COVID-19. Cell.

[CR40] Mick E, Kamm J, Pisco AO (2020). Upper airway gene expression reveals suppressed immune responses to SARS-COV-2 compared with other respiratory viruses. Nat Commun.

[CR41] Mo S, Dai L, Wang Y (2021). Comprehensive analysis of the systemic transcriptomic alternations and inflammatory response during the occurrence and progress of COVID-19. Oxid Med Cell Longev.

[CR42] Monit C, Morris ER, Ruis C (2019). Positive selection in dNTPase SAMHD1 throughout mammalian evolution. Proc Natl Acad Sci U S A.

[CR43] Mosharaf MP, Reza MS, Kibria MK (2022). Computational identification of host genomic biomarkers highlighting their functions, pathways and regulators that influence SARS-COV-2 infections and drug repurposing. Sci Rep.

[CR44] Müller JA, Groß R, Conzelmann C (2021). SARS-COV-2 infects and replicates in cells of the human endocrine and exocrine pancreas. Nat Metab.

[CR45] Nag P, Sadani K, Mukherji S (2020). Diagnosing COVID-19: the disease and tools for detection. ACS Nano.

[CR46] Newman AM, Liu CL, Green MR (2015). Robust enumeration of cell subsets from tissue expression profiles. Nat Methods.

[CR47] Ng DL, Granados AC, Santos YA (2021). A diagnostic host response biosignature for COVID-19 from RNA profiling of nasal swabs and blood. Sci Adv.

[CR48] Oliviero A, de Castro F, Coperchini F (2021). COVID-19 pulmonary and olfactory dysfunctions: is the chemokine CXCL10 the common denominator?. Neuroscientist.

[CR49] Ong EZ, Chan YFZ, Leong WY (2020). A dynamic immune response shapes COVID-19 progression. Cell Host Microbe.

[CR50] Pan Y, Zhang D, Yang P (2020). Viral load of SARS-COV-2 in clinical samples. Lancet Infect Dis.

[CR51] Pei L, Fukutani KF, Tibúrcio R (2021). Plasma metabolomics reveals dysregulated metabolic signatures in HIV-associated immune reconstitution inflammatory syndrome. Front Immunol.

[CR52] Qi M, Liu B, Li S (2021). Construction and investigation of competing endogenous rna networks and candidate genes involved in SARS-COV-2 infection. Int J Gen Med.

[CR53] Ritchie ME, Phipson B, Wu D (2015). Limma powers differential expression analyses for RNA-sequencing and microarray studies. Nucleic Acids Res.

[CR54] Sajuthi SP, DeFord P, Li Y (2020). Type 2 and interferon inflammation regulate SARS-COV-2 entry factor expression in the airway epithelium. Nat Commun.

[CR55] Salazar-Mather TP, Hokeness KL. Cytokine and chemokine networks: pathways to antiviral defense. Curr Top Microbiol Immunol. 2006; 18. 10.1007/978-3-540-33397-5_2 16570855

[CR56] Schultze JL, Aschenbrenner AC (2021). COVID-19 and the human innate immune system. Cell.

[CR57] Shaath H, Vishnubalaji R, Elkord E, Alajez NM (2020). Single-cell transcriptome analysis highlights a role for neutrophils and inflammatory macrophages in the pathogenesis of severe COVID-19. Cells.

[CR58] Shi Y, Wang Y, Shao C (2020). COVID-19 infection: the perspectives on immune responses. Cell Death Differ.

[CR59] Smith N, Goncalves P, Charbit B (2021). Distinct systemic and mucosal immune responses during acute SARS-COV-2 infection. Nat Immunol.

[CR60] Stephenson E, Reynolds G, Botting RA (2021). Single-cell multi-omics analysis of the immune response in COVID-19. Nat Med.

[CR61] Taminau J, Lazar C, Meganck S, Nowé A (2014). Comparison of merging and meta-analysis as alternative approaches for integrative gene expression analysis. ISRN Bioinforma.

[CR62] Tavassolifar MJ, Moghadasi AN, Esmaeili B (2020). Redox imbalance in CD4+ T cells of relapsing-remitting multiple sclerosis patients. Oxid Med Cell Longev.

[CR63] Tavassolifar MJ, Changaei M, Salehi Z (2021). Redox imbalance in Crohn’s disease patients is modulated by Azathioprine. Redox Rep.

[CR64] Thair SA, He YD, Hasin-Brumshtein Y (2020). Transcriptomic similarities and differences in host response between SARS-COV-2 and other viral infections. medRxiv.

[CR65] Thair SA, He YD, Hasin-Brumshtein Y (2020). Transcriptomic similarities and differences in host response between SARS-COV-2 and other viral infections. iScience.

[CR66] Tibshirani R (1997). The lasso method for variable selection in the cox model. Stat Med.

[CR67] Unterman A, Sumida TS, Nouri N (2022). Single-cell multi-omics reveals dyssynchrony of the innate and adaptive immune system in progressive COVID-19. Nat Commun.

[CR68] Vanderbeke L, Van Mol P, Van Herck Y (2021). Monocyte-driven atypical cytokine storm and aberrant neutrophil activation as key mediators of COVID-19 disease severity. Nat Commun.

[CR69] Walsh C, Hu P, Batt J, Santos C (2015). Microarray meta-analysis and cross-platform normalization: integrative genomics for robust biomarker discovery. Microarrays.

[CR70] Wang E, Erjavec S, Tejeda CI, Christiano A (2019). 085 Autoantigen screening in C3H/HeJ mouse model of alopecia areata revealed high antigenicity of melanocyte-associated antigen epitopes. J Invest Dermatol.

[CR71] Williamson EJ, Walker AJ, Bhaskaran K (2020). Factors associated with COVID-19-related death using OpenSAFELY. Nature.

[CR72] Wölfel R, Corman VM, Guggemos W (2020). Virological assessment of hospitalized patients with COVID-2019. Nature.

[CR73] Wu YY, Wang SH, Wu CH (2020). In silico immune infiltration profiling combined with functional enrichment analysis reveals a potential role for naïve B cells as a trigger for severe immune responses in the lungs of COVID-19 patients. PLoS ONE.

[CR74] Xu G, Qi F, Li H (2020). The differential immune responses to COVID-19 in peripheral and lung revealed by single-cell RNA sequencing. Cell Discov.

[CR75] Yamamoto N, Yamamoto R, Ariumi Y (2021). Does genetic predisposition contribute to the exacerbation of covid-19 symptoms in individuals with comorbidities and explain the huge mortality disparity between the east and the west?. Int J Mol Sci.

[CR76] Yang Y, Shen C, Li J (2020). Exuberant elevation of IP-10, MCP-3 and IL-1ra during SARS-COV-2 infection is associated with disease severity and fatal outcome. J Allergy Clin Immunol.

[CR77] Zhang Y, Parmigiani G, Johnson WE (2020). ComBat-seq: batch effect adjustment for RNA-seq count data. NAR Genomics Bioinforma.

[CR78] Zhou R, To KK-W, Wong Y-C (2020). Acute SARS-COV-2 infection impairs dendritic cell and T cell responses. SSRN Electron J.

[CR79] Zhou Y, Zhang J, Wang D (2021). Profiling of the immune repertoire in COVID-19 patients with mild, severe, convalescent, or retesting-positive status. J Autoimmun.

[CR80] Zhu L, Yang P, Zhao Y (2020). Single-cell sequencing of peripheral mononuclear cells reveals distinct immune response landscapes of COVID-19 and influenza patients. Immunity.

