# Supplementary material for: Identifying novel host-based diagnostic biomarker panels for COVID-19: a whole-blood/nasopharyngeal transcriptome meta-analysis
Source: Mol Med. 2022 Aug 3;28:86. doi: 10.1186/s10020-022-00513-5 (PMC9347150; doi:10.1186/s10020-022-00513-5)
Supplement: Supplementary file 1 — Additional file 1: Fig. S1. PCA plots for batch effect removal using ComBat-seq in Whole Blood samples: The PCA plots show the samples of RNA-seq datasets before (A) and after (B) batch effect removal. Fig. S2. PCA plots for batch effect removal using ComBat-seq in nasopharyngeal samples: The PCA plots show the samples of RNA-seq datasets before (A) and after (B) batch effect removal. [file 10020_2022_513_MOESM1_ESM.docx]

**Identifying novel host-based diagnostic biomarker panels for COVID-19: a whole-blood/ nasopharyngeal transcriptome meta-analysis**

Samaneh Maleknia^1^, Mohammad Javad Tavassolifar^1^, Faezeh Mottaghitalab^1^, Mohammad Reza Zali^2^, Anna Meyfour^1*^

1. Basic and Molecular Epidemiology of Gastrointestinal Disorders Research Center, Research Institute for Gastroenterology and Liver Diseases, Shahid Beheshti University of Medical Sciences, Tehran, Iran

2. Gastroenterology and Liver Diseases Research Center, Research Institute for Gastroenterology and Liver Diseases, Shahid Beheshti University of Medical Sciences, Tehran, Iran

***Corresponding author**

Anna Meyfour, Research Institute for Gastroenterology and Liver Diseases, Shahid Beheshti University of Medical Sciences, Arabi Ave., Daneshjoo Blvd., Velenjak, Tehran, Iran. Postal Code: 1985717413, Tel: +98 21 22432521. Email: [a.meyfour@sbmu.ac.ir](mailto:a.meyfour@sbmu.ac.ir)


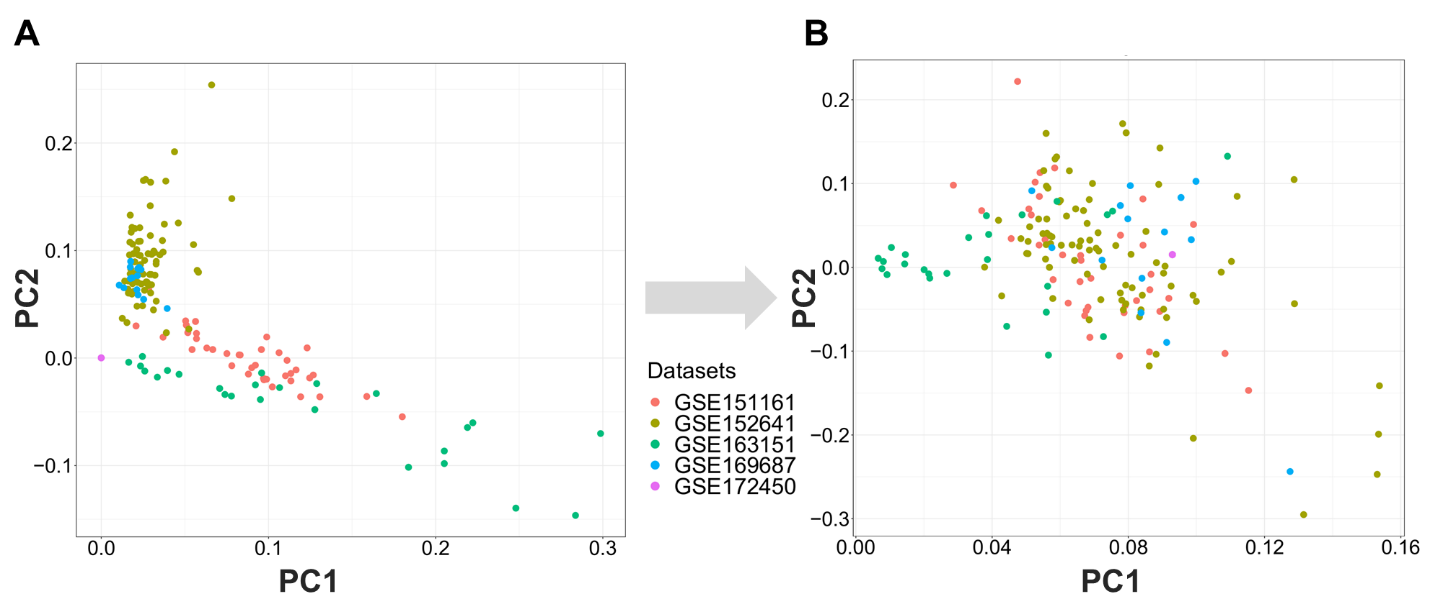


**Fig. S1 PCA plots for batch effect removal using ComBat-seq in Whole Blood samples:** The PCA plots show the samples of RNA-seq datasets before (A) and after (B) batch effect removal.


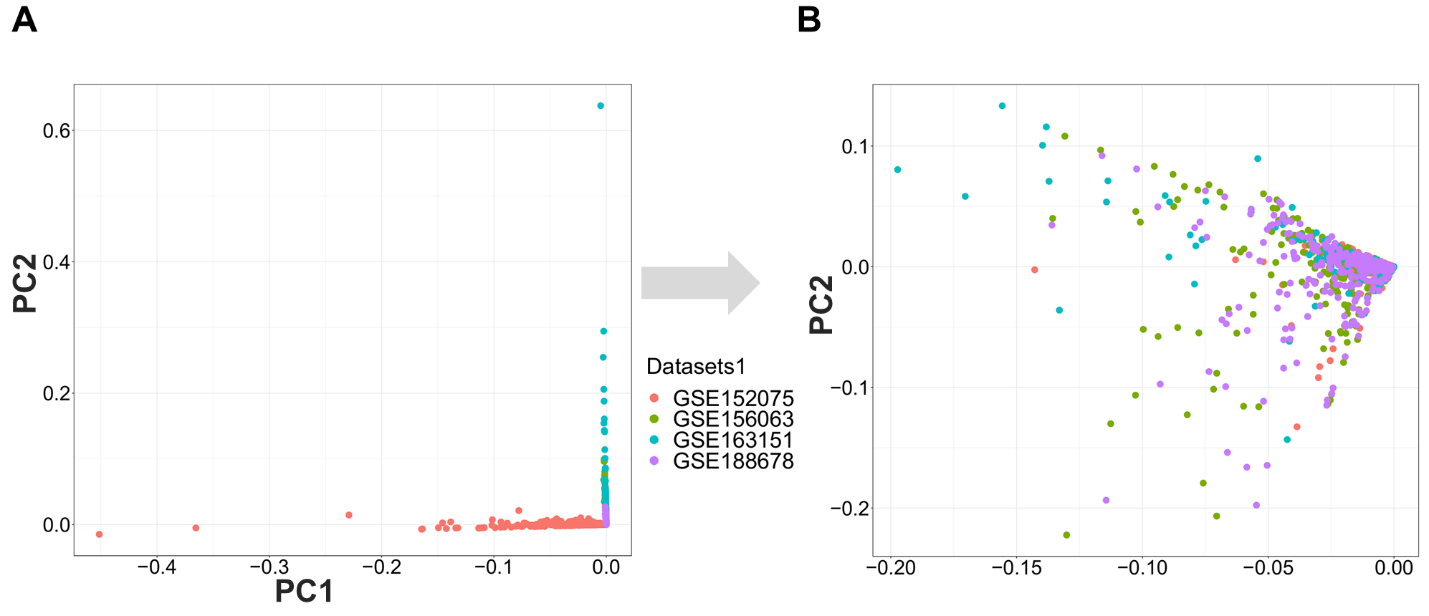


**Fig. S2 PCA plots for batch effect removal using ComBat-seq in nasopharyngeal samples:** The PCA plots show the samples of RNA-seq datasets before (A) and after (B) batch effect removal.
